# A CD6-targeted antibody-drug conjugate as a potential therapy for T cell–mediated disorders

**DOI:** 10.1172/jci.insight.172914

**Published:** 2023-12-08

**Authors:** Lingjun Zhang, Liping Luo, Jin Y. Chen, Rupesh Singh, William M. Baldwin, David A. Fox, Daniel J. Lindner, Daniel F. Martin, Rachel R. Caspi, Feng Lin

**Affiliations:** 1 Department of Inflammation and Immunity, Lerner Research Institute, Cleveland, Ohio, USA.; 2 Cole Eye Institute, Cleveland Clinic, Cleveland, Ohio, USA.; 3 Division of Rheumatology and Clinical Autoimmunity Center of Excellence, University of Michigan, Ann Arbor, Michigan, USA.; 4 Department of Translational Hematology and Oncology Research, Taussig Cancer Institute, Cleveland Clinic, Cleveland, Ohio, USA.; 5 Laboratory of Immunology, National Eye Institute, NIH, Bethesda, Maryland, USA.

**Keywords:** Autoimmunity, T cells

## Abstract

The selective targeting of pathogenic T cells is a holy grail in the development of new therapeutics for T cell–mediated disorders, including many autoimmune diseases and graft versus host disease. We describe the development of a CD6-targeted antibody-drug conjugate (CD6-ADC) by conjugating an inactive form of monomethyl auristatin E (MMAE), a potent mitotic toxin, onto a mAb against CD6, an established T cell surface marker. Even though CD6 is present on all T cells, only the activated (pathogenic) T cells vigorously divide and thus are susceptible to the antimitotic MMAE-mediated killing via the CD6-ADC. We found CD6-ADC selectively killed activated proliferating human T cells and antigen-specific mouse T cells in vitro. Furthermore, in vivo, whereas the CD6-ADC had no significant detrimental effect on normal T cells in naive CD6-humanized mice, the same dose of CD6-ADC, but not the controls, efficiently treated 2 preclinical models of autoimmune uveitis and a model of graft versus host disease. These results provide evidence suggesting that CD6-ADC could be further developed as a potential therapeutic agent for the selective elimination of pathogenic T cells and treatment of many T cell–mediated disorders.

## Introduction

Pathogenic T cells cause many diseases, including most autoimmune diseases and graft versus host disease (GVHD) (1). Selectively targeting these pathogenic T cells while sparing the normal T cells and other tissues is the holy grail of therapeutics development in modern medicine. So far, pan-immunosuppressive drugs such as corticosteroids are used to control T cell–associated inflammatory conditions, with unsatisfactory clinical efficacy and many severe adverse effects (2).

It is well established that pathogenic T cells, once activated by auto- or alloantigens, start to rapidly proliferate, causing tissue damage while the other normal T cells remain quiescent. Selectively targeting the proliferating T cells while leaving the quiescent T cells alone would be an effective strategy to develop new drugs for diseases mediated by pathogenic T cells. Mitotic toxins selectively kill actively dividing cells and have been successfully used for the treatment of cancer since tumor cells generally grow aggressively (3). Because normal tissue cells such as hair follicles and intestinal epithelial cells also proliferate under physiological conditions, these normal cells are also affected, manifesting in the adverse reactions commonly seen with these chemotherapies (4, 5). To selectively eliminate the pathogenic proliferating T cells, chemotherapeutic mitotic toxins need to be delivered directly into T cells.

Antibody-drug conjugates (ADCs) are emerging as promising cancer treatments. Developed by conjugating a potent toxin onto a monoclonal antibody (mAb) specific for a cancer cell surface antigen, the toxin is delivered selectively into the target cancer cells by the mAb after it binds to the surface antigen and is internalized to kill the cancer cells without significant toxicity to other tissues (6). Monomethyl auristatin E (MMAE), a potent mitotic toxin, is the payload in several FDA-approved ADCs, and it kills the actively dividing cancer cells by blocking the polymerization of tubulin, rapidly inducing apoptosis (7). These cancer cells and pathogenic T cells have one feature in common — both of them are actively proliferating. Therefore, this ADC approach, which has proven successful in cancer treatment, could be repurposed to selectively kill pathogenic T cells for the treatment of T cell–mediated diseases, if the mitotic toxin MMAE can be targeted into the T cells.

CD6 is an established surface marker expressed at high levels on all T cells except Tregs (8). Even though a small portion of the B cells (<1%) termed B1a cells (9) and some of the NK cells (10) are also positive for CD6, it is not detectable on other tissue cells, making it a good target candidate for T cells. We have developed CD6-knockout mice and CD6-humanized mice and showed the genetic depletion of CD6, or pharmaceutic blockage of CD6 using mAbs, protects the mice from models of T cell–mediated diseases such as multiple sclerosis (11), rheumatoid arthritis (12), and autoimmune uveitis (13).

Following an FDA-approved approach to cancer treatment, we developed a CD6-ADC by conjugating an MMAE onto a high-affinity anti-human CD6 mAb. We showed that this CD6-ADC efficiently kills malignant T cells and is effective in treating a preclinical model of T cell lymphoma (TCL) (14). In this project, we examined the killing effects of this CD6-ADC on normal human T and NK cells as well as activated human T cells and antigen-specific proliferating uveitogenic T cells in vitro. We also tested its treatment efficacies in 2 preclinical models of autoimmune uveitis using our CD6-humanized mice and a humanized model of GVHD using NOD/SCID IL-2 receptor γ^–/–^ (NSG) mice.

## Results

### Development of a CD6-ADC and nonbinding control ADC using an MMAE as the payload.

We generated the ADCs by conjugating the MMAE onto the purified anti-CD6 IgG or mouse IgG via a valine-citrulline p-aminobenzylcarbamate (VC-PAB) linker. using a commercially available kit following the protocol provided by the manufacturer. The target payload-to-antibody ratio is approximately 3:1 according to the spectroscopy analysis measuring OD_418 nm_/OD_280 nm_. The prepared CD6-ADC and control ADC were aliquoted, lyophilized, and stored in a –80°C freezer until each experiment (14).

### CD6-ADC kills proliferating T cells but not other proliferating cells that do not express CD6.

**Figure 1:**
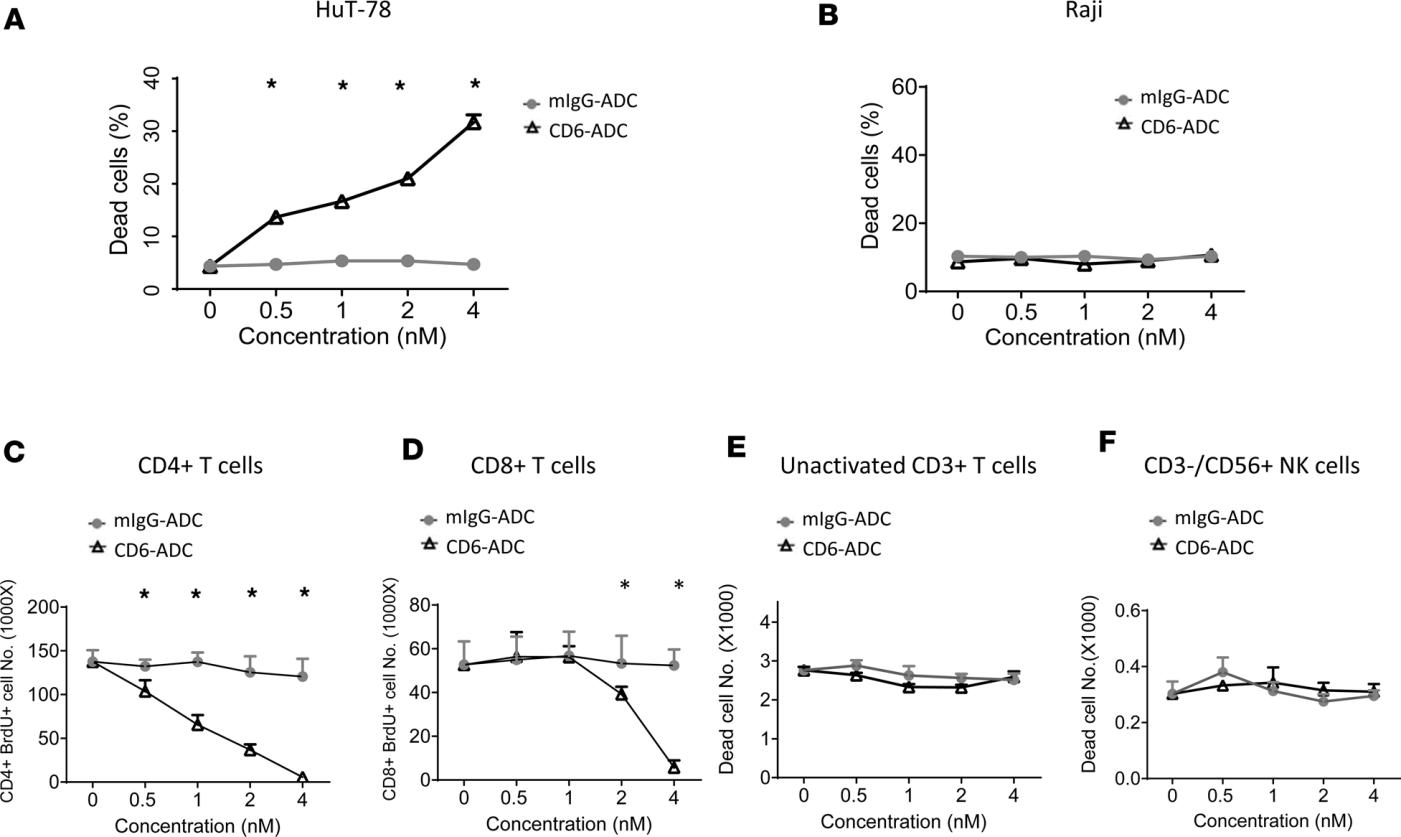
CD6-ADC selectively eliminates proliferating human T cells while sparing normal T cells and NK cells that also express CD6. CD6-ADC potently kills proliferating T cells (**A**) but not proliferating B cells (**B**) in a concentration-dependent manner. Hut-78 T cells (a human T cell line) and Raji cells (a human B cell line) were cultured with different concentrations (0, 0.5, 1, 2, 4 nM) of CD6-ADC or control ADC for 72 hours. Dead and live cells were counted after trypan blue staining using an automatic cell counter, and dead cell percentages were calculated and presented. CD6-ADC also significantly kills proliferating human primary CD4^+^ (**C**) and CD8^+^ (**D**) T cells in a concentration-dependent manner. PBMCs from healthy donors were activated and incubated with different concentrations (0, 0.5, 1, 2, 4 nM) of CD6-ADC or murine IgG–ADC (mIgG-ADC) for 5 days. BrdU was added to the culture media 16 hours before the analyses on day 5. Proliferating CD4^+^ or CD8^+^ T cells were identified by flow cytometry as CD4^+^BrdU^+^ or CD8^+^BrdU^+^ cells. CD6-ADC does not kill quiescent primary human T cells (**E**) or NK cells (**F**). PBMCs from healthy donors were cultured without activation for 3 days, with different dosages (0, 0.5, 1, 2, 4 nM) of CD6-ADC and mIgG-ADC. Numbers of dead T cells (CD3^+^) and NK cells (CD3^–^CD56^+^) were quantitated by flow cytometry after staining with a LIVE/DEAD dye. Representative data from 3 experiments. Data represent mean ± SEM. *P < 0.05. Two-way ANOVA and Bonferroni’s multiple-comparison test.

To demonstrate that our CD6-ADC kills proliferating T cells but not other proliferating cells that do not express CD6, we set up a cell killing assay using a human T cell line HuT-78 and a human B cell line Raji. Both cell lines actively divide under normal culture conditions, yet only the HuT-78 expresses CD6. These experiments showed that only the CD6-ADC, but not the control ADC, efficiently killed the proliferating T cells in a concentration-dependent manner (Figure 1A). At the same time, neither the CD6-ADC nor the control ADC had any significant detrimental effect on the proliferating Raji B cells that do not express CD6 (Figure 1B). These results indicate the CD6-ADC selectively killed proliferating T cells while sparing non–CD6-expressing cells even when they are actively dividing.

### CD6-ADC kills activated proliferating primary human T cells in vitro.

The HuT-78 cells used in the above-described studies are malignant T cells. To demonstrate that CD6-ADC efficiently kills activated normal primary human T cells, we activated primary human T cells using anti-CD3/CD28 Dynabeads, analyzed the expression levels of CD6 on CD4^+^ and CD8^+^ T cells as well as CD3^–^CD56^+^ NK cells, and assessed CD6-ADC–mediated killing. These experiments showed that CD6 expression levels on these cells were similar before and after activation (Supplemental Figure 1; supplemental material available online with this article; https://doi.org/10.1172/jci.insight.172914DS1). Again, while the control ADC did not have a significant impact on the proliferating CD4^+^ or CD8^+^ T cells in all concentrations tested, CD6-ADC markedly reduced the numbers of these proliferating T cells in a concentration-dependent manner, with the activated CD4^+^ T cells being apparently more susceptible to the killing than the CD8^+^ T cells (Figure 1, C and D). Nevertheless, at the concentration of 4 nM, the CD6-ADC eliminated almost all these activated proliferating human primary CD4^+^ and CD8^+^ T cells (Figure 1, C and D).

### CD6-ADC does not kill normal primary human T cells and NK cells in vitro.

Besides all T cells, CD6 is also present on some of the NK cells (10). To demonstrate our CD6-ADC spares normal T cells and NK cells that are quiescent but express CD6, we again set up the killing assays using primary PBMCs with 0–4 nM of CD6-ADC or control ADC. These studies showed that compared with the controls in 0 nM, neither the control ADC nor the CD6-ADC had any significant negative effect on these normal primary human T (CD3^+^) or NK cells (CD3^–^CD56^+^) even at the highest concentration tested (Figure 1, E and F). These studies provided direct evidence showing our CD6-ADC does not kill normal (quiescent) human T cells or NK cells even when they express CD6.

### CD6-ADC eliminates antigen-specific uveitogenic mouse T cells in vitro.

**Figure 2:**
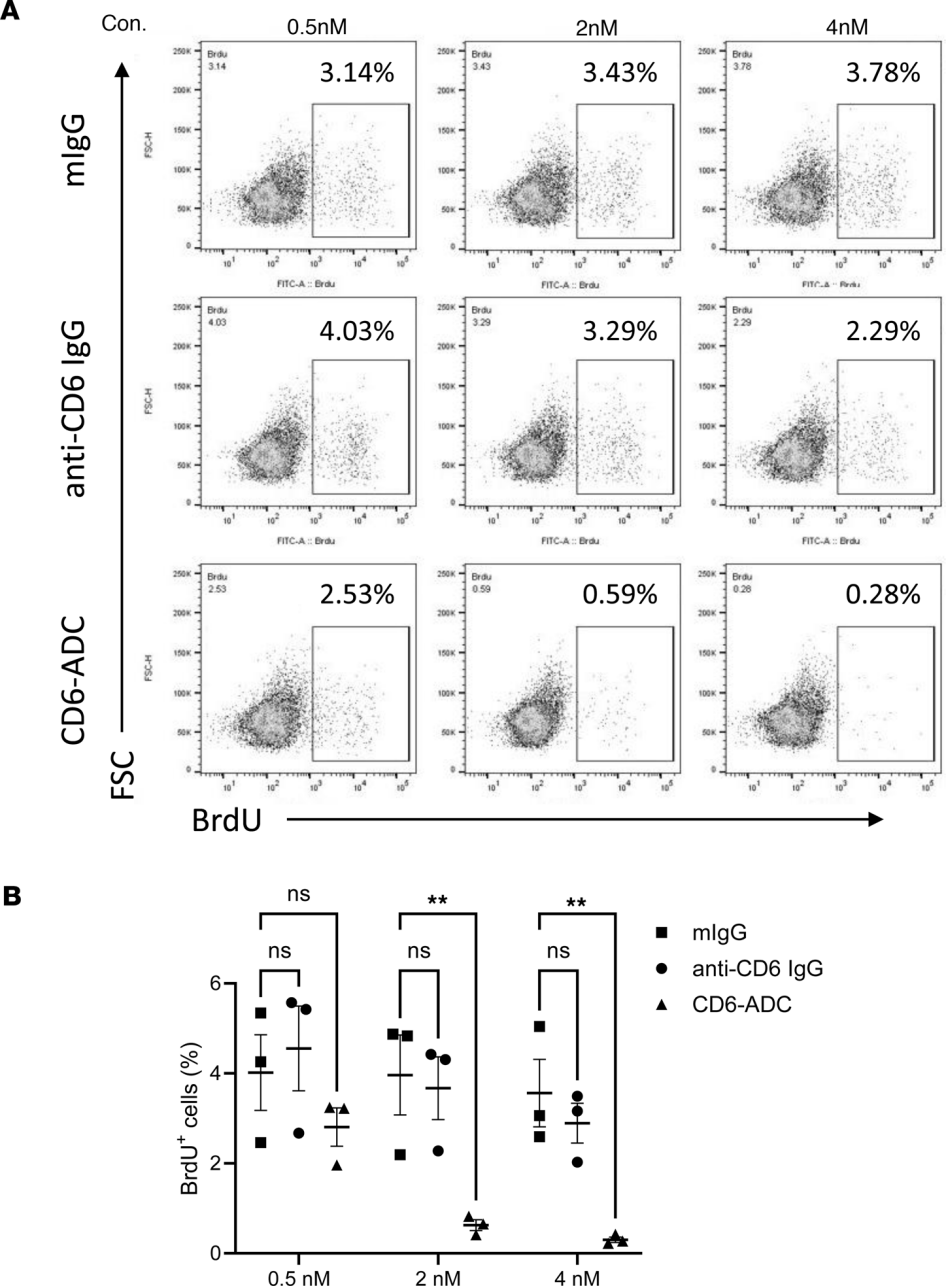
CD6-ADC kills activated antigen-specific T cells. Splenocytes from immunized mice were restimulated with 20 μg/mL of the same IRBP peptide in the presence of different concentrations (0.5, 2, 4 nM) of CD6-ADC or anti-CD6 IgG or mIgG for 3 days. BrdU was added 16 hours before the analyses. Proliferating CD4^+^ T cells after IRBP restimulation were identified as CD4^+^BrdU^+^ cells by flow cytometry. (**A**) Representative results of the CD4^+^BrdU^+^ T cells in different groups. (**B**) Summary results of the CD4^+^BrdU^+^ T cell quantification in different groups. Data represent mean ± SEM. **P < 0.01. Two-way ANOVA and Bonferroni’s multiple-comparison test.

To examine the potential of CD6-ADC in eliminating proliferating antigen-specific pathogenic T cells, we set up an antigen-specific recall assay using the splenocytes from IRBP peptide–immunized CD6-humanized mice. These experiments showed in the splenocytes analyzed, only 3%–4% of the CD4^+^ T cells were IRBP-responsive proliferating cells (BrdU^+^), which is expected from an immunized mouse. While the parent anti-CD6 mAb and the control IgG had no significant impact on the antigen-specific BrdU^+^CD4^+^ T cells at all the concentrations tested, the CD6-ADC markedly reduced the numbers of proliferating BrdU^+^CD4^+^ T cells in a concentration-dependent manner in the cultures and almost eliminated all the proliferating antigen-specific CD4^+^ T cells at the concentration of 4 nM (Figure 2). These results demonstrated CD6-ADC selectively killed the IRBP-responsive proliferating CD4^+^ T cells that are known to cause uveitis.

### A single dose of CD6-ADC suppresses the development of uveitis induced by an adoptive transfer of preactivated uveitogenic T cells.

**Figure 3:**
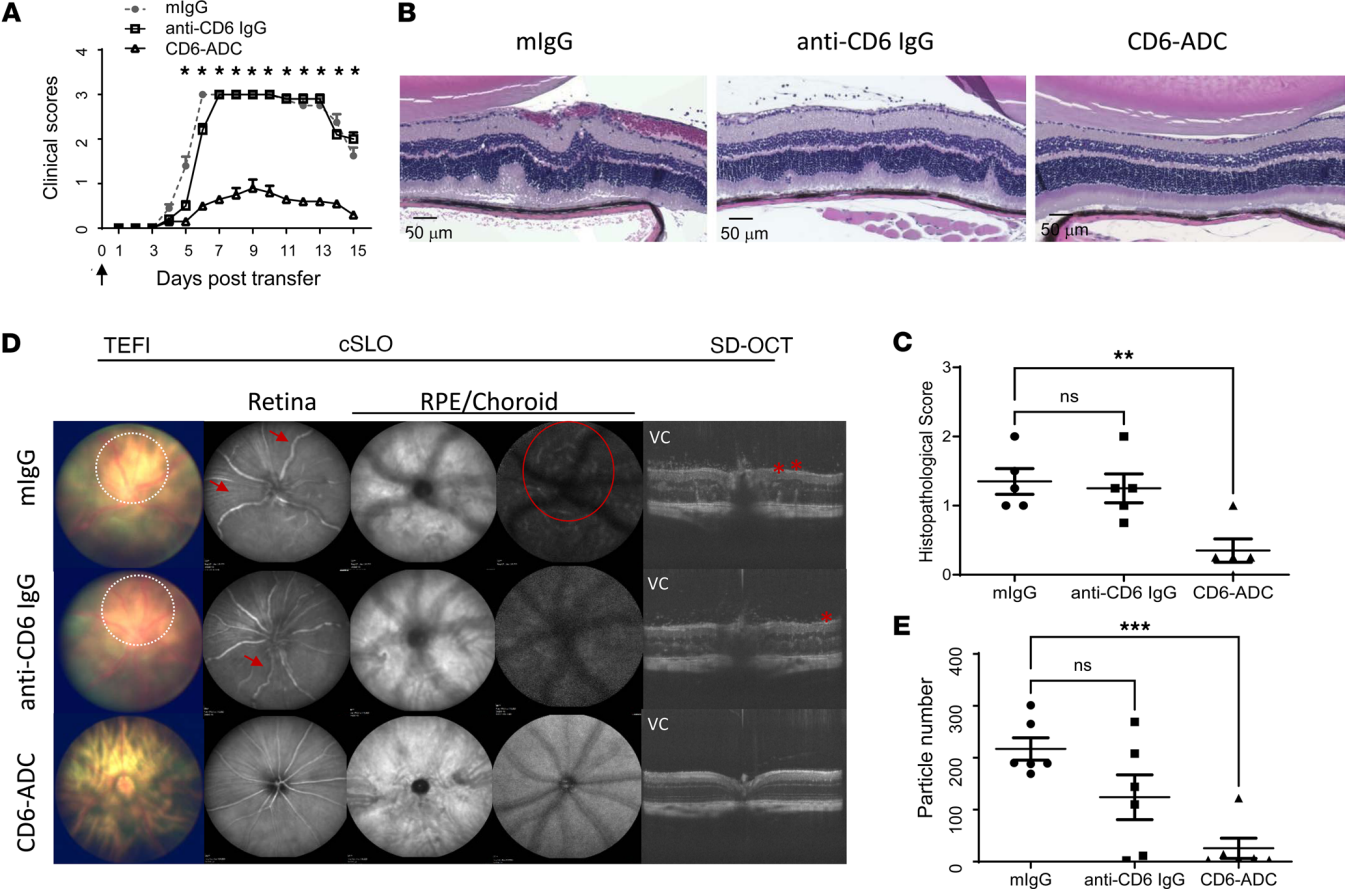
A single dose of CD6-ADC treatment significantly attenuates retinal inflammation in a passive model of EAU. Naive mice were adoptively transferred with preactivated uveitogenic T cells to induce EAU. CD6-ADC (0.5 mg/kg) or controls were given to recipient mice on the same day as the EAU induction. (**A**) Mice with CD6-ADC treatment exhibited reduced clinical score. (**B** and **C**) Representative histopathological images and scores for CD6-ADC–treated and control mice on day 15. *n* = 5 per group. (**D**) Representative of images of topical endoscopic fundus imaging (TEFI), confocal scanning laser ophthalmoscopy (cSLO), and spectral-domain optical coherence tomography (SD-OCT) in CD6-ADC–treated and control mice on day 8 after transfer. Mock-treated mice showed clear signs of uveitis including lesions surrounding the optic disc (shown with white dashed circles), enlarged and twisted blood vessels in the inner retina (arrows), blurry images, and hyperreflective lesions (red circle) in the retinal pigment epithelium (RPE)/choroid indicating edema in the retina, hyperreflective foci in the vitreous chamber (VC) indicating cell infiltrations, and folds (stars) within the retina. Many fewer abnormalities were detected in the CD6-ADC–treated mice. (**E**) Quantification of hyperreflective particles in the VC detected by SD-OCT. *n* = 6 eyes per group. Data represent mean ± SEM. *P < 0.05, **P < 0.01, ***P < 0.001. Two-way ANOVA and Bonferroni’s multiple-comparison test for clinical scores. One-way ANOVA with Dunnett’s multiple-comparison test for histological scores and the image quantification.

We then tested the efficacy of the CD6-ADC in preventing the development of uveitis induced by the adoptive transfer of preactivated uveitogenic T cells. Under the indirect ophthalmoscope, experimental autoimmune uveitis (EAU) mice treated with the control IgG showed significant intraocular inflammation with infiltrating cells, papilledema, and large chorioretinal lesions (Figure 3A). Mice treated with the parent anti-CD6 mAb had milder inflammation at the onset (day 5) but developed EAU comparable to the control group at the peak. Mice treated with the CD6-ADC showed significantly attenuated disease with only mild vasculitis and a few focal lesions. Clinical scores were assigned accordingly (Figure 3A).

Consistent with the results from the indirect ophthalmoscopy studies, later histopathological examinations of eye sections showed severe retinal damage, such as inflammatory cell infiltration and retinal folds, in the control IgG– and anti-CD6 mAb–treated mice, with limited retinal abnormalities in the CD6-ADC–treated mice (Figure 3, B and C). Similarly, in live ocular imaging examinations, large lesions (shown with circles), enlarged retinal vessels (arrows), and retinal folds (stars) were observed in the mock-treated mice but not the CD6-ADC–treated mice. Spectral-domain optical coherence tomography (SD-OCT) also revealed elevated numbers of hyperreflective foci in the vitreous chamber (VC) in the mock-treated mice, compared with those in the CD6-ADC–treated group (Figure 3, D and E). All these studies showed at the dose given, a single treatment of the CD6-ADC significantly protected the mice from retinal inflammation induced by the uveitogenic T cells. Neither the parent anti-CD6 IgG nor the control IgG at the same dose, however, offered protection, although the treatment with the parent anti-CD6 IgG slightly delayed the disease onset (Figure 3).

### CD6-ADC reverses the progress of uveitis induced by active immunization.

**Figure 4:**
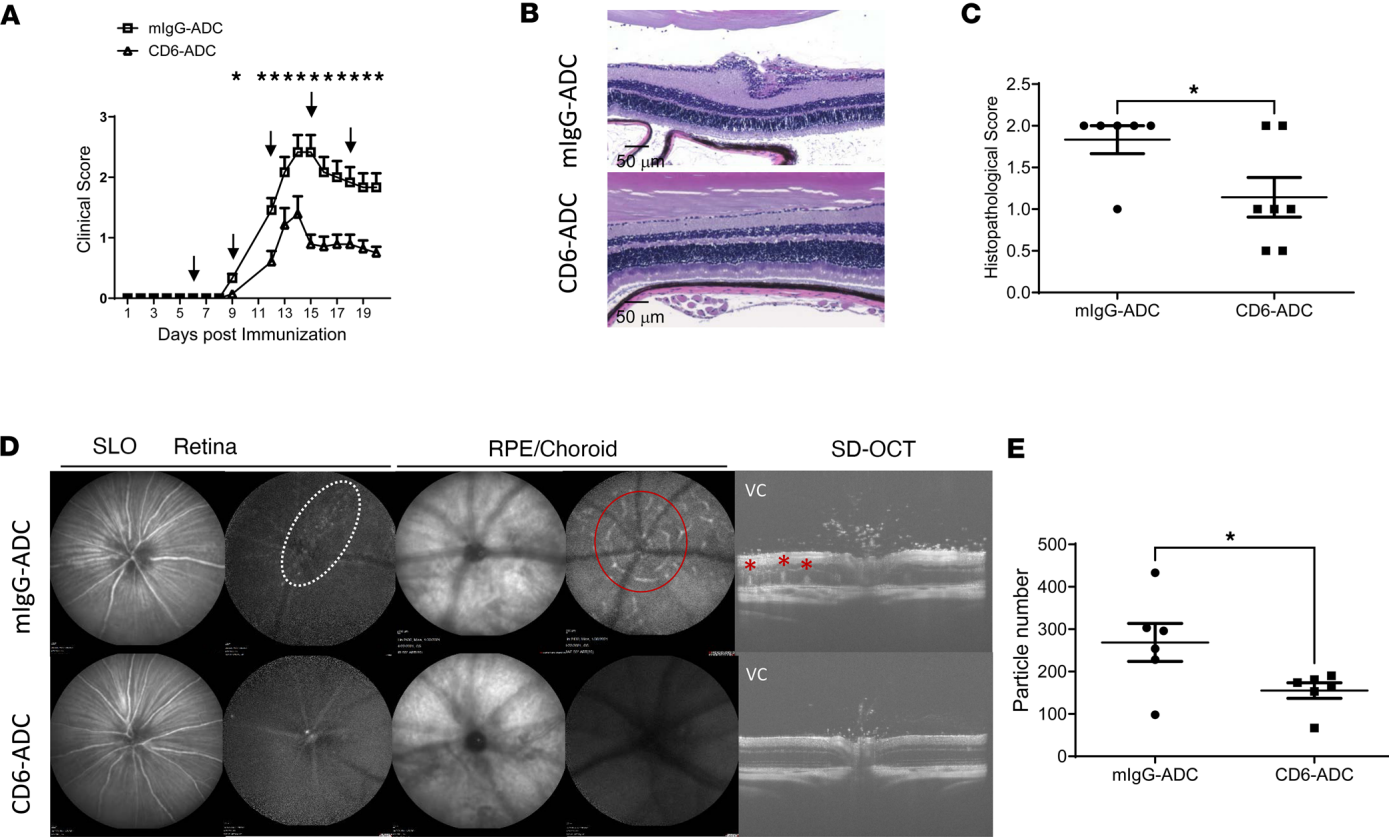
CD6-ADC treatments attenuate retinal inflammation in an active model of EAU. CD6-humanized mice were immunized with IRBP peptide to induce EAU. Six days after immunization when signs of uveitis were identified, the mice were treated with the same dose of CD6-ADC or mIgG-ADC (0.5 mg/kg) every 3 days, and the development of uveitis was monitored daily by indirect ophthalmoscopy. Mice with CD6-ADC treatments exhibited significantly reduced clinical scores compared with mice with IgG-ADC treatments (**A**). Representative histopathological images of the retinas of the CD6-ADC–treated and mock-treated mice on day 20 (**B**) and the histopathological scores (**C**). *n* = 6 for mIgG-ADC group and *n* = 7 for CD6-ADC group. Representative images of confocal scanning laser ophthalmoscope (cSLO) and spectral-domain optical coherence tomography (SD-OCT) in the CD6-ADC–treated and mock-treated mice on day 14 after immunization, which showed significant numbers of uveitis features and infiltrating cells adjacent to retinal vessels (white dashed oval) and in the vitreous chamber (VC), retinal lesions (red circle), and folds (stars) in the mock-treated but not the CD6-ADC–treated mice (**D**). Quantification of hyperreflective particles in the VC (**E**). *n* = 6 eyes per group. Data represent mean ± SEM. *P < 0.05. Two-way ANOVA and Bonferroni’s multiple-comparison test for clinical scores. Two-tailed *t* test for histological scores and the image quantification.

Data from the above-described passive uveitis model suggested the potential of CD6-ADC in preventing relapses of autoimmune uveitis. Additionally, we tested the treatment efficacy of the CD6-ADC in an autoimmune uveitis model induced by active immunization with progressive disease. We found mice with CD6-ADC treatment developed significantly attenuated uveitis, with reduced clinical scores (Figure 4A). In addition, we examined the mouse eyes by SD-OCT and cSLO on day 14. These imaging examinations also showed many more typical features of EAU, including cell infiltrations (shown with white circle), lesions (red circle), and retinal folds (stars), in the mock-treated mice than the CD6-ADC–treated mice (Figure 4, D and E). At the end of the experiment, histopathological analyses of the retinal sections also indicated fewer retinal lesions and significantly lower scores in the CD6-ADC–treated mice than the mock-treated mice (Figure 4, B and C).

### CD6-ADC treatment eliminates activated human T cells in a preclinical model of GVHD.

**Figure 5:**
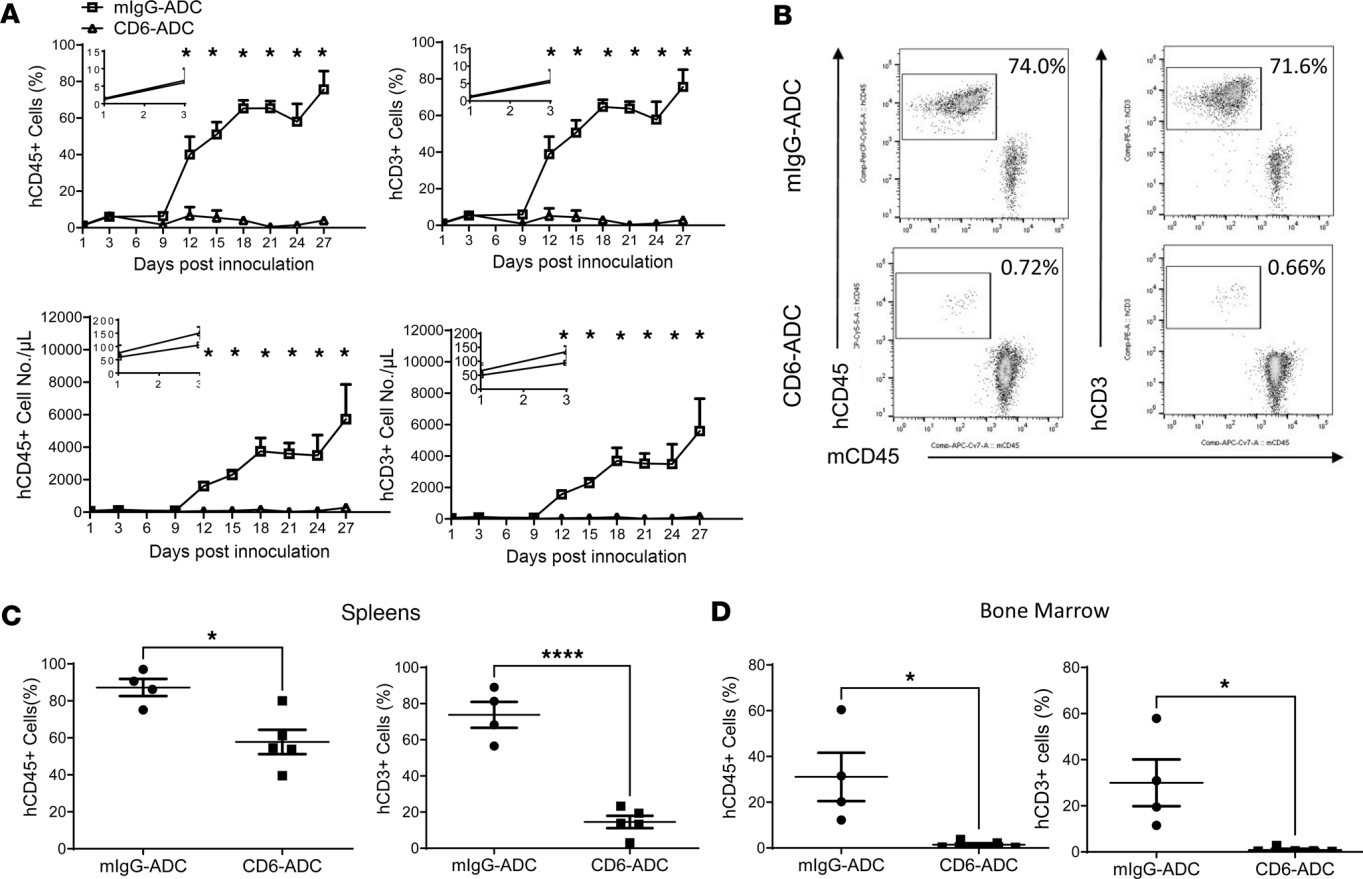
CD6-ADC treatments potently eliminate the pathogenic T cells in a preclinical model of GVHD. Irradiated NSG mice were adoptively transferred with human PBMCs. Three days later when increased numbers of human CD3^+^ T cells were detected in the circulation by flow cytometry, mice were treated with the same dose of CD6-ADC or control ADC (0.5 mg/kg) by i.p. injection every 3 days. The frequencies and absolute numbers of circulating human CD45^+^ leukocytes and CD3^+^ T cells were quantitated by flow cytometry. Small inserts in each figure showed increasing human CD45^+^ and CD3^+^ cells in the first 3 days after GVHD induction (**A**). *n* = 5 per group. Representative flow results showing massive amounts of circulating human CD45^+^ leukocytes in the mock-treated mice yet only minimally detectable circulating human CD45^+^ leukocytes in the CD6-ADC–treated mice on day 27 (**B**). Percentages and numbers of human CD45^+^ leukocytes and CD3^+^ T cells were also significantly reduced in the CD6-ADC–treated mouse spleens (**C**) and bone marrow (**D**). Data represent mean ± SEM. *P < 0.05, ****P < 0.0001. Two-way ANOVA and Bonferroni’s multiple-comparison test for **A**. Two-tailed *t* test for **C** and **D**.

**Figure 6:**
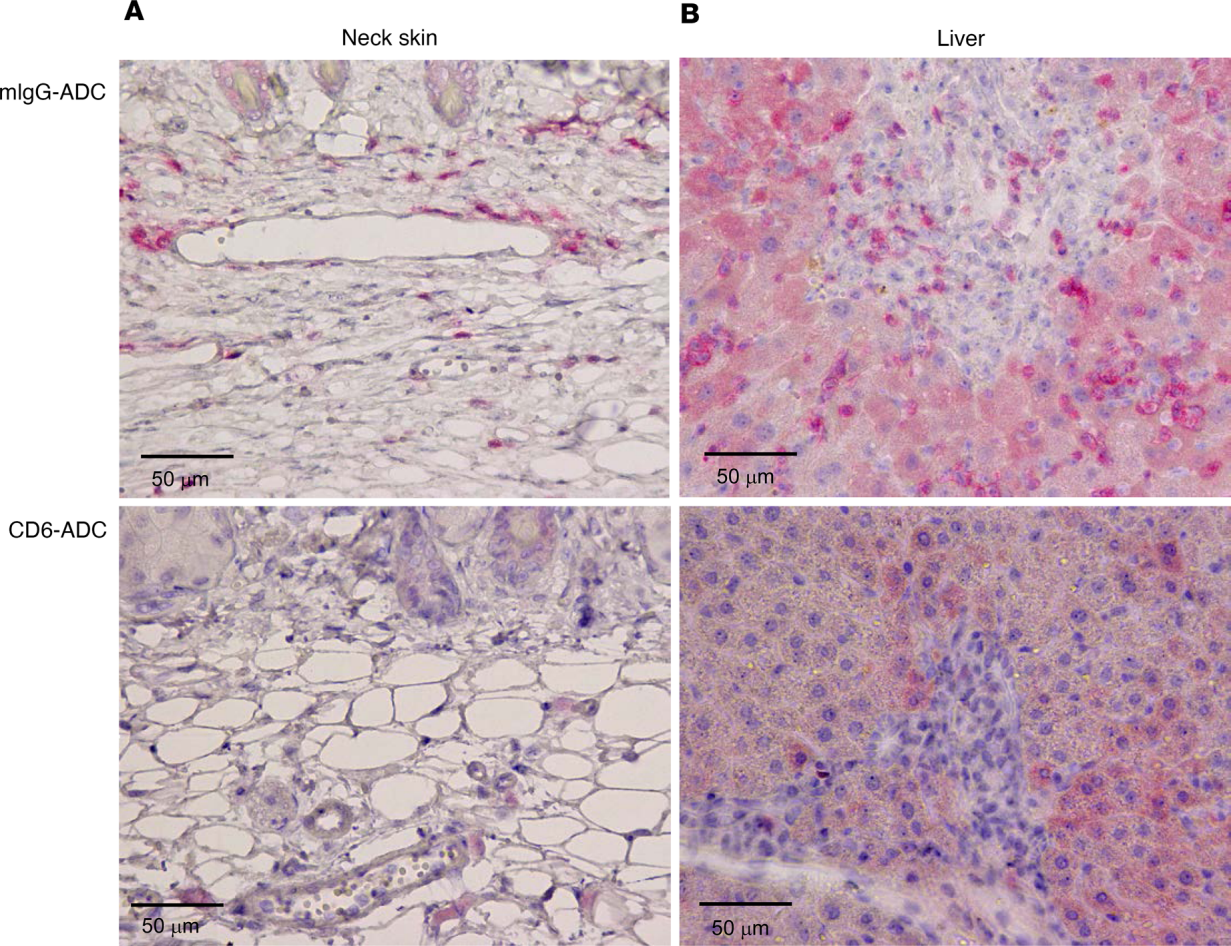
CD6-ADC treatments substantially attenuated T cell infiltration in the skin and liver. Neck skin and liver samples were collected on day 27 and processed for immunohistochemical staining of human CD3 to identify the infiltrating pathogenic T cells. Large numbers of CD3^+^ T cells (red) in the dermis of neck skin adjacent to capillaries and veins (**A**), and in the portal triad and sinusoids of the liver (**B**), were detected in the mIgG-ADC–treated mice but not in CD6-ADC–treated mice.

To determine the potential of the CD6-ADC in treating other T cell–mediated disorders, we next studied an established preclinical GVHD model. We found that while human (h) CD45^+^ and hCD3^+^ cells expanded significantly in mice treated with the control ADC after GVHD induction, which reached more than 80% of all circulating white blood cells on day 27 with the majority of the hCD45^+^ cells being hCD3^+^ T cells, there were few circulating hCD45^+^ or hCD3^+^ cells detectable in the blood from mice treated with CD6-ADC (results of day 27 mock- versus CD6-ADC–treated mice in percentages and absolute numbers/μL: CD45^+^ cells: 75.73% ± 9.95% vs. 4.34% ± 1.64%, 5,727 ± 2,143 vs. 270 ± 97; CD3^+^ cells: 75.63% ± 9.40% vs. 2.98% ± 1.6%, 5,596 ± 2,056 vs. 155 ± 64) (Figure 5, A and B). Similar results were found in the spleen and bone marrow samples on day 27 (Figure 5, C and D). Histopathological examinations of different tissues verified these hematological analysis results, showing that CD6-ADC treatment markedly reduced hCD3^+^ cell infiltration in the dermis of the neck skin, adjacent to capillaries and veins (Figure 6A), and in the portal triad and sinusoids of the liver (Figure 6B). In addition, substantial structural damages were found in organs of mice with mIgG-ADC treatments, such as the irregular arrangement and even loss of keratinocytes and hepatocytes, but not in the CD6-ADC–treated mice (Figure 6). All these experiments showed that treatment of CD6-ADC substantially eliminated the pathogenic human T cells and attenuated GVHD in mice, suggesting that the CD6-ADC can be used to treat T cell–mediated disorders such as GVHD in additional to autoimmune uveitis.

## Discussion

In a closely related work, we identified CD6 as a potential therapeutic target for malignant T cells and demonstrated that the CD6-ADC developed by conjugating the mitotic toxin MMAE onto a high-affinity anti-CD6 mAb is highly effective in treating a preclinical model of TCL (14). In this project, we showed the CD6-ADC selectively killed proliferating nonmalignant T cells activated by specific autoantigens or by CD3/CD28 cross-linking without significantly affecting normal T cells and NK cells that also express CD6 or other proliferating cells that do not express CD6. More importantly, the CD6-ADC was highly effective in treating 2 preclinical models of autoimmune uveitis and a humanized model of GVHD, in all of which antigen-specific T cells cause the pathology. These data demonstrate the potential of the CD6-ADC as a drug candidate for treating diseases in which pathogenic T cells are causally involved in the pathogenesis besides TCLs.

More than 70 mAbs have been FDA approved for the treatment of a variety of diseases (15). In the treatment of cancers, therapeutic mAbs kill tumor cells through mechanisms such as antibody-dependent cellular cytotoxicity and complement-dependent cytotoxicity. However, many tumor cells are resistant to the direct cytotoxic effects of mAbs. To address these deficiencies, ADCs, which are mAbs connected by a specified linkage to potent cytotoxic payloads, are emerging as a significant advance in the use of the mAbs to treat cancer (6). The extremely potent toxins are delivered by mAbs specific for antigens on tumor cells, resulting in the selective killing of these malignant cells. To minimize the nonspecific cytotoxic effects of the toxins used in the ADCs, these toxins are usually developed in their inactive form and conjugated to the mAb through a cleavable linker. Only after the mAb binds to tumor cells, and is internalized, will the linker be cleaved, releasing the activated toxins to selectively kill the tumor cells from within (6). As demonstrated by the 12 ADCs already approved by the FDA for cancer treatment and the more than 100 additional ADCs currently in clinical trials for tumor immunotherapy, this approach is successful. For example, brentuximab vedotin is an FDA-approved ADC developed by conjugating an inactivated form of MMAE onto an anti-CD30 mAb (16). In patients with lymphoma expressing CD30, brentuximab vedotin binds to tumor cells through the anti-CD30 mAb and is internalized into the lysosomes. The acidic environment within the lysosome then cleaves the VC-PAB linker to release the activated MMAE to the proliferating CD30^+^ lymphoma cells by blocking the polymerization of tubulin and inducing apoptosis. Besides brentuximab vedotin, polatuzumab vedotin, an FDA-approved ADC targeting CD79b for treating B cell lymphoma (17), and enfortumab vedotin, an FDA-approved ADC targeting nectin-4 for treating urothelial cancer (18), also utilize the same linker and MMAE as the payload, demonstrating the clinical safety and efficacy of this approach for ADC development. Inspired by the clinical successes of these ADCs, we used the same linker and payload for our CD6-ADC development and demonstrated that it potently kills malignant T cells (14).

In principle, this ADC approach for treating cancers could also be employed to selectively ablate autoreactive T cells, which like tumor cells actively divide. If the latent MMAE can be target-delivered into T cells, and activated inside, it should selectively kill the proliferating (autoreactive) T cells from within while leaving quiescent normal T cells and other tissue cells (proliferating or not) unaffected. Indeed, an ultimate goal of treating autoimmune diseases mediated by pathogenic T cells is to selectively target these autoreactive T cells while sparing the normal quiescent T cells and other tissue cells. Our CD6-ADC appeared to achieve this goal in 3 ways. First, CD6 is almost exclusively expressed on T cells; the other cells known to express CD6 are B1a cells, which account for less than 1% of the total B cells, and some NK cells. Second, MMAE, being an antimitotic drug, kills actively proliferating cells. Even though CD6 is present on all T cells, under normal conditions, resting T cells are not actively proliferating; therefore, these T cells are not sensitive to the MMAE-mediated killing. On the contrary, the actively dividing auto- or alloreactive pathogenic T cells become victims of the CD6-ADC–mediated killing as indicated in all our in vitro and in vivo studies. Third, the anti-CD6 mAb used to develop the CD6-ADC does not deplete T cells but is rapidly internalized into T cells after binding to CD6 (11, 14).

To the best of our knowledge, only 1 other ADC is under active development for treating autoimmune diseases. This ADC is generated by conjugating amanitin, an RNA polymerase II inhibitor, onto a mAb against CD45, which is present on all leukocytes, including T cells, B cells, NK cells, eosinophils, basophils, monocytes, macrophages, and neutrophils. Published meeting abstracts (19) showed this ADC is highly effective in treating models of multiple sclerosis (MS) and GVHD as well as inflammatory arthritis. Although this CD45-targeted ADC demonstrates the applications of ADC are indeed not limited to tumor immunotherapy and can be extended to autoimmune disease treatment, it is substantially different from our CD6-ADC. First, unlike CD45, which is expressed in all leukocytes and some stem cells, CD6 is primarily expressed on T cells. Thus, unlike the nonspecific cytotoxic effects of the CD45-directed ADC targeting all leukocytes, our proposed therapy targets only T cells, and should, therefore, not lead to systemic immunosuppression and the related severe side effects. Second, the payload used in the CD45-targeted ADC, amanitin, kills both proliferating and quiescent cells while the MMAE used in our CD6-ADC is a mitotic toxin that kills only proliferating cells. By combining the T cell selectivity of the anti-CD6 mAb and the proliferating cell selectivity of the payload MMAE, our CD6-ADC should have a much better safety profile and fewer side effects by selectively targeting only proliferating T cells. Indeed, all the treated mice in our studies tolerated the CD6-ADC well without any apparent issues. Of course, more detailed and focused studies are warranted to rigorously establish the safety profile of the CD6-ADC in the future.

We previously reported the parental anti-CD6 mAb used in the CD6-ADC development is effective in treating mouse models of autoimmune diseases such as MS (11) and rheumatoid arthritis (RA) (12) by suppressing T cell responses without depleting the CD6^+^ T cells. The CD6-ADC, in theory, should have significantly greater treatment efficacy than its parental “naked” mAb because of the potent payload conjugated. Indeed, in our previous reports (11, 12), when given at approximately 4 mg/kg (~100 μg/mouse), the anti-CD6 mAb was very effective in treating models of MS and RA. In the treatment experiments described in this report, however, we found at the dose given, 0.5 mg/kg (~12 μg/mouse), CD6-ADC significantly suppressed the development of uveitis (Figure 3A) after the adoptive transfer of preactivated uveitogenic T cells, but the same dose of the “naked” anti-CD6 mAb only delayed the development of uveitis and moderately attenuated retina inflammation in the treated mice within the first week of uveitis development. These data indicated the CD6-ADC has a significantly heightened treatment efficacy (Figure 3) and much lower level of effective dose than the parent “naked” anti-CD6 mAb in treating autoimmune diseases, which could lead to many benefits, such as reduced costs and decreased potential side effects. The synergetic effects between the “naked” anti-CD6 mAb and the potent payload could also contribute to the observed treatment potency of the CD6-ADC.

In addition to many autoimmune diseases such as autoimmune uveitis, GVHD is another disorder primarily mediated by activated pathogenic T cells. GVHD occurs in most patients after allogeneic bone marrow (BM) transplantation (20), which is the last resort for diseases such as sickle cell anemia and many hematologic malignancies. Despite the understanding that activated and expanded donor T cells damage the host tissues to cause GVHD, currently available therapeutic options are limited, are unsatisfactory, and have severe side effects. We employed a xeno-GVHD model, which is commonly used to evaluate potential drug candidates for treating GVHD in humans. It has been established that in this model, after the adoptive transfer of human PBMCs, the T cells are activated and then proliferate to cause tissue damage (21–23). In this humanized model of GVHD, we found that the CD6-ADC, but not the control ADC, efficiently killed the expanding human T cells in mice. This was true even at a low dose of 0.5 mg/kg after the human T cells were activated in vivo. This led to significantly reduced numbers of human T cells in the circulation and, consequently, markedly attenuated pathology in multiple organs such as liver, spleen, and skin (Figure 5A).

Of course, when patients are infected, pathogen-specific T cells are activated and start to proliferate. If these patients are still under the treatment of the CD6-ADC, their pathogen-specific T cells will also be sensitive to the CD6-ADC–mediated killing, which could lead to opportunistic infections. To mitigate these complications in these cases, the CD6-ADC treatment regimen can be halted until antibiotics and/or antiviral drugs are administrated to help the patients control the invading pathogens.

In summary, the CD6-ADC selectively kills proliferating pathogenic T cells and is highly effective in treating 2 preclinical models of autoimmune uveitis as well as a humanized model of GVHD even when given at a low dose. These intriguing results suggest that the CD6-ADC holds promise as a drug for treating pathogenic T cell–mediated disorders, including but not limited to diseases like autoimmune uveitis, MS, RA, GVHD, and transplantation rejections.

## Methods

### Generation of the CD6-ADC and control ADC.

MMAE was conjugated onto a purified mouse anti-human CD6 IgG (UMCD6) (11) or control mouse IgG via the VC-PAB linker using a kit (CellMosaic Inc.) by following the manufacturer-provided protocol. The targeted drug-to-antibody ratio of the resultant products was estimated by measuring OD_418 nm_/OD_280 nm_.

### Human T cell line (HuT-78) and B cell line (Raji) killing assays.

Human T cell line HuT-78 (ATCC) or B cell line Raji (ATCC) were seeded at 40,000 cells/well in a 96-well plate in complete RPMI 1640 medium containing 0, 0.5, 1, 2, or 4 nM of CD6-ADC or control ADC. After 6 hours of incubation, cells were washed and cultured in complete RPMI 1640 medium for another 72 hours, and then live and dead cells in each well were counted using a Countess automatic cell counter (Invitrogen) after trypan blue staining.

### Human primary activated T cell killing assay.

Commercially purchased PBMCs from healthy donors were seeded in a U-bottom, 96-well plate at a final concentration of 5 × 10^5^ cells/mL in RPMI 1640 medium supplemented with hIL-2 (100 U/mL, PeproTech). T cells were either unactivated or activated with Dynabeads coupled with anti-CD3 and anti-CD28 antibodies (Thermo Fisher Scientific, catalog 11131D) at a ratio of 1:1. They were then incubated with 0, 0.5, 1, 2, or 4 nM of CD6-ADC or control ADC for 5 days. A total of 10 μM of BrdU was added to the culture media 16 hours before harvesting the cells. The number of PBMCs were counted under a microscope, and the frequencies of CD3^+^, CD4^+^, and CD8^+^ T cells were detected by anti-human CD3, CD4, and CD8 mAbs (BioLegend, catalog 981004, 980812, 344714) followed by flow cytometry. To assess the T cell proliferation, BrdU incorporation was detected by BrdU flow kit (BD Biosciences, catalog 559619) and analyzed using flow cytometry (BD LSRFortessa).

### Human primary normal T cell and NK cell killing assays.

Killing assays were set up again using normal PBMCs as described above but without the T cell–activating Dynabeads. After incubation with different concentrations of CD6-ADC and control ADC for 5 days, cells were stained with the LIVE/DEAD dye (Thermo Fisher Scientific) and anti-human CD3 and CD56 mAbs (BioLegend, catalog 981004, 362504) to identify the T cells (CD3^+^) and NK (CD3^–^CD56^+^) cells, which were then analyzed by flow cytometry.

### Antigen-specific mouse T cell killing assay.

Each of the CD6-humanized mice (DBA/1 background, 8 to 12 weeks old, generated in our lab) (11) was subcutaneously immunized with a 200 μL complete Freund’s adjuvant (Difco Laboratories, Inc.) containing 200 μg of the uveitogenic IRBP_161-180_ peptide (SGIPYIISYLHPGNTILHVD; GenScript) and 250 μg *Mycobacterium tuberculosis* H37Ra (Difco Laboratories, Inc.). Splenocytes from the immunized CD6 humanized mice were isolated 12 days later. A total of 4 × 10^5^ splenocytes were then restimulated for 3 days with 20 μg/mL IRBP_161-180_ peptide in the presence of 0.5, 2, and 4 nM of CD6-ADC, anti-CD6 IgG, or mouse IgG, respectively, in RPMI 1640 medium. BrdU was added to the culture media 16 hours before collecting the cells. Cells were stained with anti-mouse CD4 (BioLegend, catalog 100430) and anti-BrdU mAb (BD Biosciences) followed by analyses of BrdU incorporation in the CD4^+^ T cells using a flow cytometer.

### CD6-ADC treatments of active and passive models of EAU.

The inductions of active and passive models of EAU were performed as previously described (13). For the treatment of active EAU, immunized mice were first treated 6 days after immunization by intraperitoneal injections of 0.5 mg/kg of CD6-ADC, anti-CD6 IgG, or control IgG when retinal hyperreflective foci were detected by cSLO (HRA2/Spectralis, Heidelberg Engineering). For the treatment of passive EAU, the recipient mice were treated only once after the adoptive transfer of preactivated uveitogenic T cells with the same dose of the different agents. The development of EAU was monitored daily using an indirect ophthalmoscope and was assigned clinical scores of 0 to 4 according to previously published criteria (13).

### Ocular imaging and histopathological analyses.

Ocular imaging was performed as previously described (13, 24). In brief, under anesthesia and pupil dilation, mice were imaged by cSLO and SD-OCT (Bioptigen, Inc.). For SD-OCT images, numbers of hyperreflective particles in VC areas were further analyzed and quantified using ImageJ software (NIH). At the end of EAU studies, whole eyes were collected for H&E staining. The sections were assigned histopathological scores of 0–4 according to previously published criteria based on the inflammatory infiltration of, and structural damage to, the retina (13, 24).

### CD6-ADC treatment of a model of GVHD.

NSG mice (The Jackson Laboratory, 8 weeks) were irradiated (2 Gy) and were given 3 × 10^6^ human PBMCs intravenously by tail vein injection (25). Peripheral blood cells were collected every 3 days after induction and analyzed by flow cytometry analyses after staining with anti-mouse CD45, anti-human CD45, and anti-human CD3 mAbs (BioLegend, catalog 103116, 368504, 981004). Treatments of 0.5 mg/kg CD6-ADC and mIgG-ADC were administered intraperitoneally every 3 days starting from day 3, when flow cytometry detected increased numbers of human CD45^+^ leukocytes in the peripheral blood, an indication of ongoing GVHD development. After 27 days, splenocytes and cells from BM were isolated, and the percentages of hCD45^+^ and hCD3^+^ cells in total white blood cells (mCD45^+^ and hCD45^+^ cells) were detected by a flow cytometer. The neck skin and liver were harvested, fixed in 10% formalin solution, embedded in paraffin, and stained with H&E. hCD3^+^ T cells were detected by immunohistochemical staining.

### Statistics.

Student’s *t* test (2 tailed) was used to compare means of 2 experimental groups, and for multiple comparisons 2-way ANOVA followed by Bonferroni’s posttest was used. Data are presented as the mean ± SEM; *P* values of less than 0.05 were considered significant. All statistical analyses were performed using GraphPad Prism 8.0.

### Study approval.

All animals were used in compliance with the guidelines approved by the Cleveland Clinic Institutional Animal Care and Use Committee. PBMC collection and usage were approved by the Institutional Review Boards of Cleveland Clinic.

### Data availability.

The underlying values for data presented in the graphs and supplemental figure are available in the Supporting Data Values file. All other additional data are available from the corresponding author upon request.

## Author contributions

LZ, LL, JYC, and RS did experiments, analyzed data, and edited the manuscript. WMB, DAF, DJL, DFM, and RRC supervised the studies, discussed the results, and edited the manuscript. FL conceived the study, designed the experiments, and prepared the manuscript.

## Supplementary Material

Supplemental data

Supporting data values

## Version 1 11/02/2023

In-Press Preview

## Version 2 12/08/2023

Electronic publication
