# Supplementary material for: A CD6-targeted antibody-drug conjugate as a potential therapy for T cell–mediated disorders
Source: JCI Insight. 2023 Dec 8;8(23):e172914. doi: 10.1172/jci.insight.172914 (PMC10795824; doi:10.1172/jci.insight.172914)
Supplement: Supplemental data [file jciinsight-8-172914-s113.pdf]

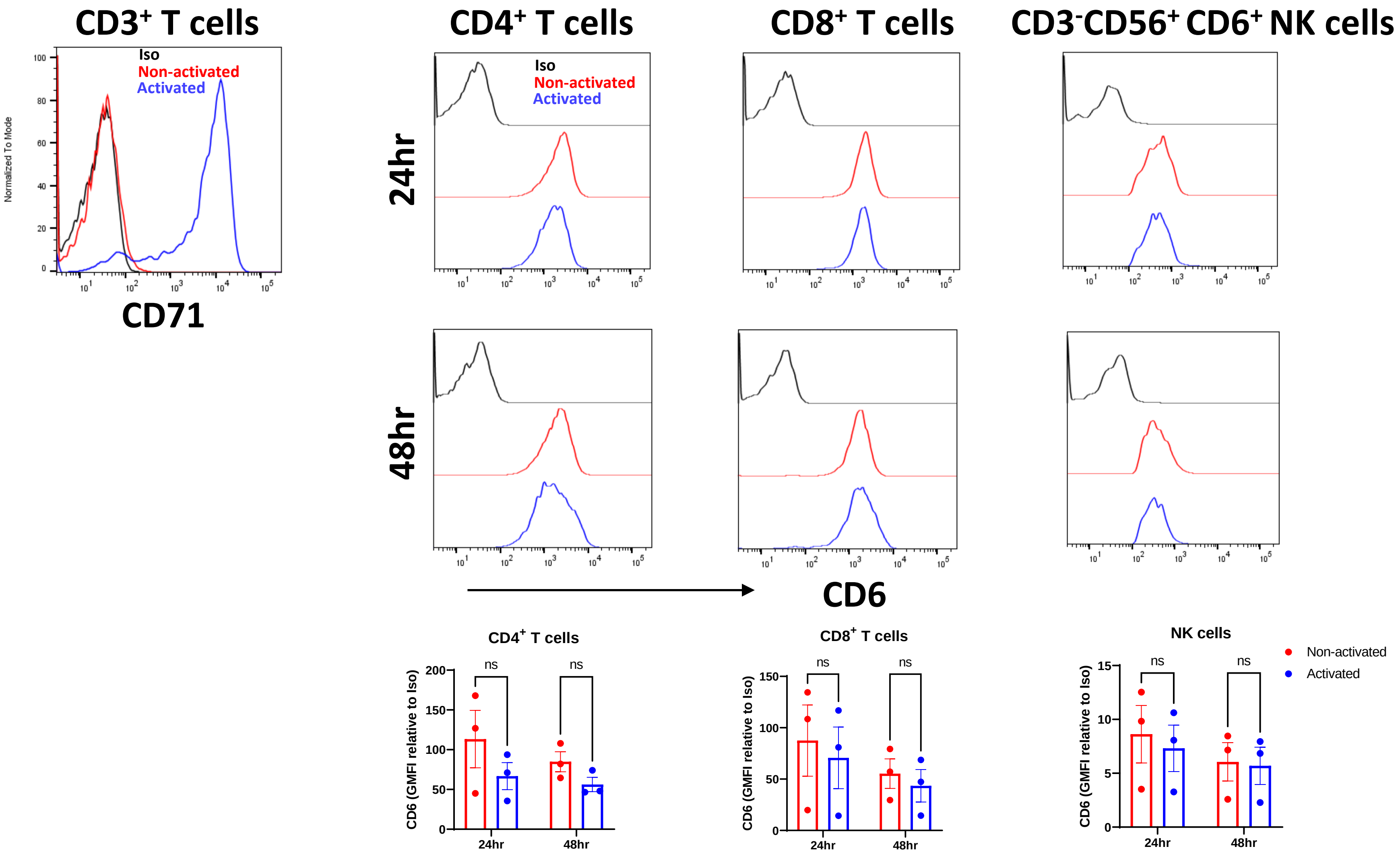

Suppl Fig. 1 CD6 expression on non-activated and activated T cells, and CD6⁺ natural killer (NK) cells. Healthy donor PBMCs were cultured and activated with Human T-Activator Dynabeads CD3/CD28 beads and 100U/ml human IL-2 for 24 and 48 hours. T cell activation was confirmed by CD71 expression on CD3⁺ T cell surface. CD6 expression was examined by a 488-conjugated goat anti-CD6 antibody, represented by histogram from one healthy donor and bar graph from three healthy donors. Two-way ANOVA analysis was applied. ns, non significant.
